# Advances in Meniscal Tissue Engineering

**DOI:** 10.1155/2012/420346

**Published:** 2011-10-26

**Authors:** Umile Giuseppe Longo, Mattia Loppini, Francisco Forriol, Giovanni Romeo, Nicola Maffulli, Vincenzo Denaro

**Affiliations:** ^1^Department of Orthopaedic and Trauma Surgery, Campus Bio-Medico University of Rome, Via Álvaro del Portillo 200, 00128 Rome, Italy; ^2^Centro Integrato di Ricerca (CIR), Università Campus Bio-Medico di Roma, Via Álvaro del Portillo 21, 00128 Rome, Italy; ^3^University CEU San Pablo, 28003 Madrid, Spain; ^4^Centre for Sports and Exercise Medicine, Barts and The London School of Medicine and Dentistry, Mile End Hospital, 275 Bancroft Road, London E1 4DG, UK

## Abstract

Meniscal tears are the most common knee injuries and have a poor ability of healing. In the last few decades, several techniques have been increasingly used to optimize meniscal healing. Current research efforts of tissue engineering try to combine cell-based therapy, growth factors, gene therapy, and reabsorbable scaffolds to promote healing of meniscal defects. Preliminary studies did not allow to draw definitive conclusions on the use of these techniques for routine management of meniscal lesions. We performed a review of the available literature on current techniques of tissue engineering for the management of meniscal tears.

## 1. Introduction

The menisci of the knee are two fibrocartilaginous C-shaped discs interposed between the femoral and tibial joint surfaces. They provide shock absorption, stabilization, lubrication, load distribution, and joint filler supplying femoral-tibial incongruity [1, 2]. Traumatic lesions of the menisci are common and induce changes in biomechanical behaviour of the joint affecting the load distribution and contact stresses [3]. The healing process of torn menisci depends on their morphologic features. Each meniscus consists of outer vascular part and inner avascular part. The vascular supply is an important factor to determine the potential healing of meniscal tears [4]. Therefore, lesions of the outer one-third of the meniscus are believed to have the greatest capacity for repair. Meniscal tears are usually located in the inner avascular part of the meniscus and are not able to heal spontaneously. Several strategies to repair and replace meniscus have been proposed, but only few of them have been shown to be effective [5–9].

Depending on the type of lesion, surgical approaches include total or subtotal meniscectomy, transplantation, and repair [10]. As the fibrocartilaginous tissue of the meniscus presents a limited regenerative capacity, new approaches are required to improve meniscal healing. In the last few decades, several emerging strategies, including growth factors, gene therapy, and application of mesenchymal stem cells (MSCs), have been proposed to increase healing of a damaged meniscus by tissue-engineered constructs. Tissue engineering is based on a combination of cells, growth factors, and scaffolds able to stimulate the meniscal healing [11, 12].

We performed a review of the available literature on current techniques of tissue engineering for the management of meniscal tears.

## 2. Cells Transplantation

Human menisci are populated by different cell types that might respond differently to various stimuli released from the matrix [13, 14]. Cell-based therapy has significantly contributed to develop tissue-engineering strategies consisting of cells-scaffold constructs able to promote healing in an avascular environment [15]. Autologous fibrochondrocytes are one of the cell types used in meniscal repair. Fibrochondrocytes are able to proliferate and produce new extracellular matrix (ECM) [16]. The amount of glycosaminoglycans (GAGs) produced by fibrochondrocytes from the inner avascular part is more than the amount produced from a peripheral fibrous location when seeded into a porous collagen scaffold [17, 18]. Although these findings are encouraging, the application of autologous fibrochondrocytes in meniscal tissue engineering is limited by the difficulty to harvest a sufficient number of cells.

An alternative cell type used to promote the healing of meniscal lesions is the articular chondrocyte [19, 20]. Peretti et al. [19] described a porcine chondrocyte model where implantation of such cells was performed in the avascular part of the meniscus, using an allogenic scaffold seeded with autologous chondrocytes, showing that these chondrocytes were able to heal a meniscal tear [19]. Another potential cell therapy approach is represented by MSCs. These pluripotent cells are able to differentiate into specific therapeutic cell types (developmental plasticity) [21–23]. 

The effects of extrinsic stimuli (biochemical, physical, and mechanical) from the microenvironment, within a cell/scaffold combination, are a promising alternative for repairing large meniscal defects [24]. Several studies confirm production of abundant extracellular matrix around the cells, restoring a meniscal-like tissue in the avascular zone [25–28]. In particular, the combination of growth factors and mesenchymal stem cells within scaffold implants increased proteoglycan and/or collagen synthesis [26, 28, 29].

The effect of load on all these different cell types becomes an interesting field for future research. Moreover, their stimulation with the application of growth factors in combination with a mechanically loadable scaffold has been proposed as the focus of future studies.

## 3. Growth Factors

Growth factors typically act on target cells as signalling molecules, promoting cell differentiation and chondrocytic proliferation [30]. They also stimulate the synthesis and inhibit degradation of (extracellular matrix) ECM by a mechanism of downregulation of proteases [31]. Several growth factors have been demonstrated to have an effect on the healing of tears and on ECM synthesis in tissue and cell culture. In particular, transforming growth factor-*β*1 (TGF-*β*1) seems to have several regulatory activities to stimulate the production of extracellular matrix and collagen type II by meniscus cells [30, 32]. Application of this growth factor has resulted in the synthesis of specific proteoglycans to enhance both collagen and GAGs production and their biomechanical properties [33, 34]. Pangborn and Athanasiou [35] used TGF-*β*1 to have consistent effects on collagen and proteoglycan production by meniscal cells. TGF-*β*1 was applied to monolayer cultures for 3 weeks and generally showed a higher production of each ECM component. 

Fibroblast growth factor-2 (FGF-2) is another important factor found in the cartilaginous matrix. It enhances proliferation of the joint chondrocytes, mesenchymal stem cells, osteoblast, and adipocytes. In addition, FGF can also maintain the ability of any cell types to differentiate [36, 37]. Recently, FGF-2 has been vectored with recombinant adenoassociated virus (rAAV) [38]. Histology demonstrated enhanced cell proliferation and expression of the *α*-smooth muscle actin (*α*-SMA) contractile marker, but it did not significantly enhance the synthesis of major extracellular matrix components or DNA contents. 

Other authors have identified basic fibroblast growth factor (bFGF) as effective at stimulating extracellular matrix production in cell and tissue development. The ovine experimental model showed the presence of meniscal fibrochondrocytes responding to bFGF by proliferating and producing new extracellular matrix [16].

The insulin growth factors (IGFs), particularly IGF-1, are considered the main anabolic growth factor of articular cartilage [39, 40]. IGF-1 stimulates the synthesis of proteoglycans, collagen II, and integrins. In a recent study, the effects of three growth factors regimens was examined: basic fibroblast growth factor (bFGF) alone, bFGF plus transforming growth factor (TGF-*β*1), and IGF-1 [41]. The mixture of growth factors showed an upregulation of collagen II and aggrecans under the effects of TGF-*β*1 and IGF-1 that may be an important cellular response to mediate avascular meniscal healing. 

The induction of angiogenesis is an important factor to stimulate the poor potential healing of meniscal tears. The vascular endothelial growth factor (VEGF) may promote better healing, stimulating angiogenesis to improve the healing capacities of meniscus tissue. However, a study by Petersen et al. did not lead to satisfactory results, and the local application of VEGF did not promote meniscus healing [42]. 

Bone morphogenetic proteins (BMPs) are members of the TGF-*β* superfamily and play an important role during embryogenesis and tissue repair by their osteoinductive properties [43, 44]. BMP-2 acts as a stimulus in the differentiation of mesenchymal cell. It also presents a migratory effect in endothelial cells or smooth muscle cells, but rarely in chondrocytes. Alternatively, BMP-7 can have a function in regulating matrix homeostasi and can inhibit the degradation processes. BMP-7 acts with different chondrogenic agents and is more effective than BMP-2 for chondrogenic differentiation of MSCs [45]. Minehara et al. [46] developed a new technique for seeding chondrocytes onto solvent-preserved human meniscus using the chemokinetic effect of recombinant human bone morphogenetic protein-2 (rhBMP-2) on chondrocytes seeded into solvent-preserved human meniscus. After a 3-week incubation, a natural chemokinetic effect of rhBMP-2 promoted migration and proliferation of chondrocytes. These findings demonstrate that BMPs induce a marked cellular response to improve meniscal repair.

## 4. Preparations Rich in Growth Factors

The application of growth factors has been proven to be effective for meniscal healing. Recently, platelet-rich plasma (PRP) may be better than the use of isolated growth factors. PRP is an autologous substance rich in platelets. It is easily prepared by spinning autologous blood in a centrifuge to form a dense fibrin matrix that can be placed directly at the meniscal repair site [47, 48]. Ishida et al. reported the regenerative effects of platelet-rich plasma in a rabbit model [49]. Cultured meniscal cells were prepared to assess proliferative pattern under the exposure to PRP. Histological findings showed the healing properties of PRP in extracellular matrix synthesis and cell proliferation.

## 5. Biomaterial Used in Tissue Engineering

Tissue engineering techniques using novel scaffold materials offer potential alternatives for managing meniscal tears. An ideal scaffold should have the basic structure of the meniscus, and it should be biodegradable and bio-reabsorbable in the long term. Probably, the most important functions are the induction of cell proliferation and production of extracellular matrix, using it as a carrier for stimulatory and inhibitory growth. The structure should be strong enough to withstand the load in the joint and maintain its structural integrity without damaging the articular cartilage [15, 31, 50–52].

Several materials used to fabricate scaffolds (natural or synthetic) may be considered for application in tissue engineering of the meniscal healing. The first to be developed are natural scaffolds as periosteal tissue, perichondral tissue, collagen, small intestine submucosa, silk, and meniscus tissue itself [53].

A multilayered (tribiological), multiporous silk scaffold system to mimic native meniscus architecture and shape was described [54]. Silk constructs showed a good biocompatibility with a florid chondrogenesis as well as other tissues [54–57]. The cells (human articular chondrocytes and dermal fibroblast cells) were seeded onto the silk scaffold in association with human chondrocytes for 28 days. Histological analysis showed an increase production of GAGs and proteoglycans and a colonization of ECM similar to native tissue from fibroblasts and chondrocytes. 

Minehara et al. developed a cell-seeding technique using a solvent-preserved human meniscus as a scaffold [46]. The chondral cells were treated with recombinant human bone morphogenetic protein-2 (rhBMP-2) and cultured for 3 weeks. The histological and immunohistochemical analyses indicated that this repair tissue was mainly fibrous. Moreover, results suggest a potential application of rhBMP-2 as a natural chemokinetic factor into a scaffold for tissue engineering.

Collagen scaffolds have been also examined for tissue engineering of the meniscus. Meniscus cells seeded in these scaffolds may express alpha-smooth muscle actin (*α*-SMA) that has contractile capacities. This demonstrates the potential healing in wound contraction, but other physiological and pathological processes are still unknown [58, 59]. Mueller et al. studied collagen type I and II scaffolds seeded with canine fibrochondrocytes for 21 days [60]. Type II scaffold contained up to 50% more GAGs than type I scaffold. A limit of the collagen scaffolds may be their poor mechanical properties, as the shape of the construct cannot be varied. 

The use of synthetic polymer-based scaffolds is a novel option offering the potential of earlier healing. Stewart et al. [61] used polyglycolic acid (PGA) scaffolds seeded with ovine meniscal chondrocytes. The cells were seeded onto the PGA scaffold in the presence of platelet-derived growth factor- (PDGF-) AB, PDGF-BB, insulin-like growth factor- (IGF-) I, transforming growth factor-beta1 (TGF-*β*1), and basic fibroblast growth factor (bFGF) and evaluated after 39 days. Histological analysis of sections from ovine meniscal chondrocytes PGA scaffolds did not show any difference in GAG or collagen production between the treatment groups. However, immunohistochemical analysis demonstrated a different expression of collagen production: the production of collagen type I was increased, whereas the collagen type II was decrease at day 39 in all constructs functionalized with growth factors. A concomitant high infiltration of cells was also found. 

Another tissue-engineered strategy consists in a poly-L-lactic acid (PLLA) scaffold used in association with culture of meniscus cells and bFGF under hypoxic conditions [62]. After 4 weeks, histological evaluation demonstrated the presence of collagen and GAG, probably due to synergic effects of hypoxia and bFGF. An earlier study by Ionescu tested the effects of TGF-*β*1 as a function of age, on proliferation of bovine meniscus fibrochondrocytes (MFCs) in a polycaprolactone (PCL) cylindrical scaffold [63]. Even though the results indicated a loss of proliferation and migration capacity with aging, the addition of TGF-b showed better maintenance of overall explant properties.

## 6. Gene Therapy

Gene therapy is considered an alternative strategy to develop future protocols for tissue engineering of meniscus tissue, using viral or nonviral vectors or direct gene transfer [64, 65]. In this way, the transfer of genes used to encode healing factors is a valid technique to apply growth factors to the site of injury for extended period. The vectors most frequently used in meniscal lesion are adenovirus, adenoassociated virus (AAV), and retrovirus. Nonviral vectors are not indicated because of being less efficient, although they are less pathogenic. Viral vectors allow the insertion of genes into death cells and the production of growth factors. 

Previously, we mentioned a study where FGF-2 in association with recombinant adenoassociated virus (rAAV) vectors were used [38]. 

Goto et al. [66] developed a gene therapy strategy based on monolayer cultures of human and canine meniscal cells infected with retroviruses carrying human TGF-*β*1 cDNA or marker genes. There was an increased synthesis of collagen and proteoglycan in response to the addition of TGF-*β*1. 

Another possible technique for gene transfection is the injection of adenovirus vector encoding the hepatocyte growth factor gene (AdHGF) in cell-seeded bovine PGA scaffolds [67]. This strategy showed the formation of vascularised fibrous tissue by 2 weeks and vascularized meniscus-like tissue in 8 weeks. The authors concluded that gene transfer techniques could be used to induce blood vessel formation in tissue engineering meniscus samples.

## 7. Discussions

Application of tissue engineering is a promising alternative approach for the management of meniscus injuries. Advances in meniscal tissue engineering focus on the use of different cell sources, scaffolds, growth factors, or a combination thereof. The potential effect of cell-based therapy for meniscal tears could improve healing of lesion in the avascular zone and expand the indication for repair rather than removal. A variety of cell types such as autologous fibrochondrocytes, articular chondrocytes, and MSCs are available in large quantities into the body and can be used in tissue engineering [17–19, 28]. Of these, progenitor cells such as mesenchymal stem cells have the advantage to be easily expandable without the loss of their differentiation potential into a variety of mesenchymal tissues [68–80] including bone, tendon, cartilage, muscle, ligament, fat, and marrow strom [13, 21, 22]. Probably, the application of MSCs might be a better cell source than fibrochondrocytes for meniscus repair [81–94].

The long-term biochemical and biomechanical features of tissue engineering techniques are determined by a combination of a well-integrated cell population with a scaffold. The development of carrier scaffolds should provide mechanical stability of the meniscus, maintaining its structural integrity without damaging the articular cartilage [15, 51]. Several scaffold implants have been investigated in the management of meniscal tears [93–109]. 

The use of growth factors has been demonstrated to have an effect on the healing of tears and on ECM synthesis in tissue and cell culture [30, 61]. Direct introduction of growth factors, such as TGF-*β*, BMPs, IGF-1, FGF, and VEGF, has positively influenced the clinical outcome of the meniscal repair procedures [33, 59, 110]. Previous studies have demonstrated that the effects of TGF-*β*1 and BMPs have a better potential to help healing in tissue engineering [34, 45, 46]. The focus in this future research should be on the assessment of a mechanically loadable scaffold that retains growth factors and at the same time is degraded to allow revascularisation [111–128].

Alternatively, gene transfer techniques represent a favorable strategy for growth factor delivery, inducing vascularisation of tissue-engineered constructs [66, 67, 110]. Several viral vectors expressing therapeutic proteins such as growth factors have been investigated to assess their potential to improve remodelling and healing of meniscus. Although gene therapy is a relatively new field in tissue-engineered menisci, it is one of the treatment options in the future [129–140].

## 8. Conclusion

The importance of the meniscus in safeguarding joint function has gained a considerable interest in the recent years. The current therapeutic strategy to treat meniscal defects is partial or total meniscectomy, but this may predispose patients, especially younger individual, to early osteoarthritis changes [11, 12, 141–152]. The management of meniscal pathology to promote a healing response is considered essential in dealing with these injuries [153–168]. When possible, meniscal repair should be performed to try to maintain meniscal integrity and prevent long-term degenerative changes. 

New therapeutic strategies of meniscal replacement and tissue engineering need to be developed, but they are still at their infancies [11, 12]. The first step, the need to develop autologous grafting procedure, consists in finding the best cell source for meniscus repair, which to date seems to be the MSCs. The second step consists in fabricating an opportune biological scaffold which is able to carry cells into the meniscal lesion and to allow their differentiation, proliferation, and ECM synthesis to produce a meniscal native-like tissue. The biological activity of scaffold should be implemented through its functionalization with growth factors, such as TGF-*β*1 and BMPs [169–199].

Further research is necessary to successfully address the difficult problem of meniscal regeneration. Advancements in this field should be strongly encouraged, because of autologous grafting through either tissue engineering for repair or that complete replacement following excision represents a suitable alternative to partial or total meniscectomy or cadaveric implants.
